# Norpsilocin: freebase and fumarate salt

**DOI:** 10.1107/S2056989020004077

**Published:** 2020-03-27

**Authors:** Andrew R. Chadeayne, Duyen N. K. Pham, James A. Golen, David R. Manke

**Affiliations:** a CaaMTech, LLC, 58 East Sunset Way, Suite 209, Issaquah, WA 98027, USA; b University of Massachusetts Dartmouth, 285 Old Westport Road, North Dartmouth, MA 02747, USA

**Keywords:** crystal structure, tryptamines, indoles, hydrogen bonding

## Abstract

The solid-state structures of the naturally occurring psychoactive tryptamine norpsilocin {4-hy­droxy-*N*-methyl­tryptamine (4-HO-NMT); systematic name: 3-[2-(methyl­amino)­eth­yl]-1*H*-indol-4-ol}, C_11_H_14_N_2_O, and its fumarate salt (4-hy­droxy-*N*-methyl­tryptammonium fumarate; systematic name: bis­{[2-(4-hy­droxy-1*H*-indol-3-yl)eth­yl]methyl­aza­nium} but-2-enedioate), C_11_H_15_N_2_O^+^·0.5C_4_H_2_O_4_^2−^, are reported. The freebase of 4-HO-NMT has a single mol­ecule in the asymmetric unit joined together by N—H⋯O and O—H⋯O hydrogen bonds in a two-dimensional network parallel to the (100) plane. The ethyl­amine arm of the tryptamine is modeled as a two-component disorder with a 0.895 (3) to 0.105 (3) occupancy ratio. The fumarate salt of 4-HO-NMT crystallizes with a tryptammonium cation and one half of a fumarate dianion in the asymmetric unit. The ions are joined together by N—H⋯O and O—H⋯O hydrogen bonds to form a three-dimensional framework, as well as π–π stacking between the six-membered rings of inversion-related indoles (symmetry operation: 2 − *x*, 1 − *y*, 2 – *z*).

## Abstract 2

The solid-state structure of the ‘magic mushroom’ natural product norpsilocin is reported, as well as the synthesis and structure of its fumarate salt.

## Chemical context   

Psychoactive tryptamines, particularly psilocybin and psilocin, have recently garnered a great deal of inter­est because of their potential to treat disorders including anxiety, addiction, and depression (Johnson & Griffiths, 2017 ▸; Carhart-Harris & Goodwin, 2017 ▸). Of note, psilocybin was recently granted the ‘breakthrough therapy’ designation by the US Food and Drug Administration (Feltman, 2019 ▸). To this point, the focus of research on psychedelics in therapy has largely been on psilocybin and psilocin. Despite this focus, there are more than 200 species of ‘magic mushrooms’ containing many different psychoactive tryptamines and combinations of the same (Stamets, 1996 ▸).

The clinical effects observed for extracts of ‘magic mushrooms’ differ from those observed for pure psilocybin (Zhuk, *et al.* 2015 ▸). This indicates that the minor components of ‘magic mushrooms’ have psychoactive properties that are important, or that they work in conjunction with psilocybin as part of an entourage effect (Russo, 2011 ▸). To have a better understanding of ‘magic mushroom’ pharmacology, it is necessary to understand the properties of the minor active components. This could lead to formulations that maximize the desired activity while minimizing negative effects, optimizing the clinical experience.

Baeocystin, the monomethyl analog of psilocybin, is the second most abundant naturally occurring tryptamine found in ‘magic mushrooms’. It was first isolated from the mushroom *Psilocybe baeocystis* in 1968 (Leung & Paul, 1968 ▸), and subsequently identified in other species, approaching one third of the total tryptamine concentration. Like psilocybin, baeocystin acts as a prodrug when consumed by humans, undergoing rapid hydrolysis of the phosphate ester to afford its active metabolite – the 4-hy­droxy analog.

The prodrug psilocybin hydrolyses to the active 4-hy­droxy-*N*,*N*-di­methyl­tryptamine (4-HO-DMT), aka psilocin, and the prodrug baeocystin hydrolyses to the active 4-hy­droxy-*N*-methyl­tryptamine (4-HO-NMT), aka norpsilocin. Norpsilocin was first identified as a natural product of ‘magic mushrooms’ in 2017, and isolated as an amorphous, colorless solid (Lenz *et al.*, 2017 ▸). In 2020, norpsilocin was synthesized and isolated as a white solid in 98% purity. When tested as an agonist at the human seratonin 2a receptor, synthetic norpsilocin was as potent, if not more so, compared to psilocin (Sherwood *et al.*, 2020 ▸).
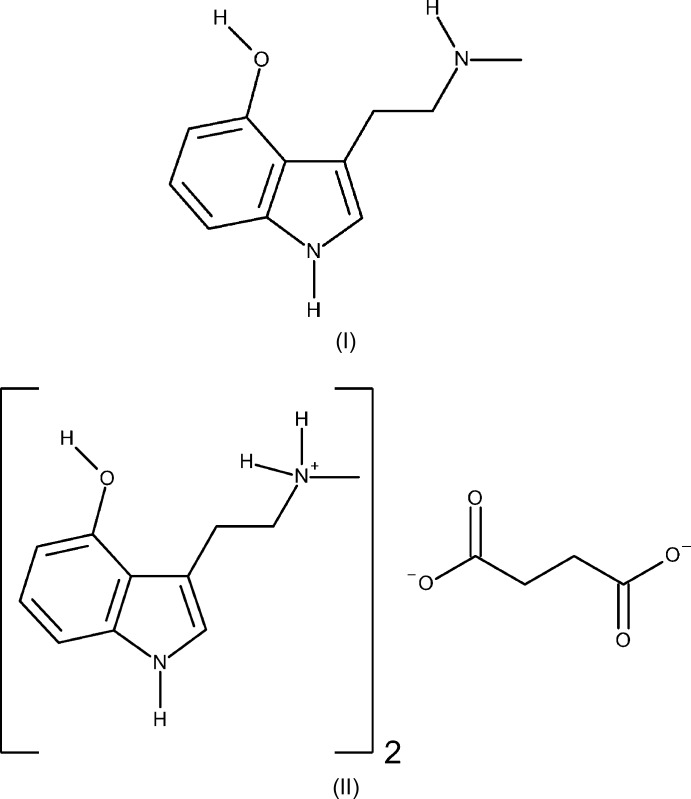


Despite rapidly growing evidence supporting psilocin/psilocybin’s potential for treating mood disorders, very little work has been done to investigate the properties of other structurally similar compounds found in magic mushrooms, *e.g.* norpsilocin/baeocystin. Although these compounds have substantial potential as drug candidates, they have undergone limited investigation because of their lack of availability in pure form and the difficulty of their purification. Crystalline solids are the most convenient and reliable chemical forms for studying, handling, and administering pure compounds. There was an unmet need for the structural characterization of norpsilocin, which is important in examining the structure–activity relationship of the psychedelic tryptamine. Herein, we report the first crystal structure of norpsilocin (I), and the first salt of norpsilocin (II) and its solid-state structure.

## Structural commentary   

**Figure 1:**
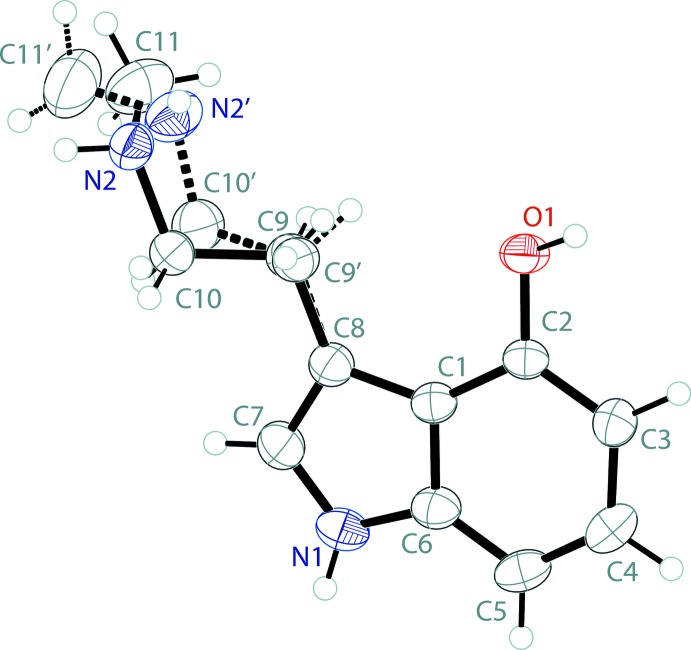
The mol­ecular structure of 4-hy­droxy-*N*-methyl­tryptamine, showing the atom labeling. Displacement ellipsoids are drawn at the 50% probability level. Dashed bonds indicate the minor occupancy disordered component in the structure.

The mol­ecular structure of the freebase of norpsilocin, 4-HO-NMT, is shown in Fig. 1 ▸. The asymmetric unit contains one full 4-hy­droxy-*N*-methyl­tryptamine (C_11_H_14_N_2_O) mol­ecule. The ethyl­amine arm (C9–C10–N2–C11) of the tryptamine is modeled as a two-component disorder with a 0.895 (3) to 0.105 (3) occupancy ratio. The rest of the discussion is restricted to the major component. The indole ring system of the tryptamine is near planar with an r.m.s. deviation from planarity of 0.015 Å. The ethyl­amine arm of the tryptamine is slightly turned, with a C7—C8—C9—C10 torsion angle of 29.3 (3)°. The C10—N2—C11 angle about the amine nitro­gen is 113.51 (15)°.

**Figure 2:**
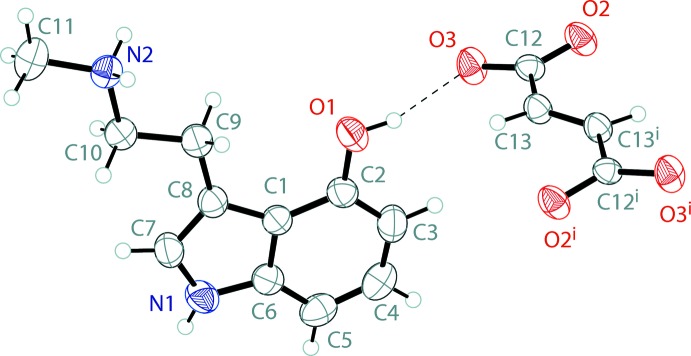
The mol­ecular structure of bis­(4-hy­droxy-*N*-methyl­tryptammonium)­fumarate, showing the atom labeling. Displacement ellipsoids are drawn at the 50% probability level. Hydrogen bonds are shown as dashed lines. Symmetry code: (i) 1 − *x*, −*y*, 2 − *z*.

The mol­ecular structure of the fumarate salt of norpsilocin is shown in Fig. 2 ▸. The asymmetric unit contains one full 4-hy­droxy-*N*-methyl­tryptammonium (C_11_H_15_N_2_O^+^) cation and one half of a fumarate (C_4_H_2_O_4_^2–^) dianion, with the other half generated by inversion. The indole ring system of the tryptamine is near planar with an r.m.s. deviation from planarity of 0.009 Å. Unlike the freebase, the ethyl ammonium arm resides in the same plane as the indole. The planarity of all of the non-hydrogen atoms of the tryptamine is demonstrated with an r.m.s. deviation from planarity of only 0.043 Å. The C10—N2—C11 angle about the ammonium nitro­gen is 114.20 (14)°. The fumarate itself is also near planar, with an r.m.s. deviation from planarity of 0.050 Å. The carboxyl­ate unit of the fumarate is delocalized, with C—O distances of 1.2488 (18) and 1.2553 (18) Å.

## Supra­molecular features   

The tryptamine mol­ecules of the freebase of norpsilocin are held in an infinite two-dimensional network parallel to the (100) plane through a series of N—H⋯O and O—H⋯N hydrogen bonds (Table 1 ▸). The phenol O—H hydrogen bonds with the nitro­gen of the methyl­amine of an inversion-related tryptamine mol­ecule (symmetry operation: −*x* + 1, −*y* + 1, −*z* + 1) to form a dimer. The indole N—H shows an inter­molecular hydrogen bond with the phenol oxygen of another tryptamine mol­ecule (symmetry operation: *x*, −*y* + 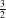, *z* − 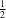), joining the dimers into two-dimensional sheets. The packing of 4-HO-NMT is shown in Fig. 4 ▸ *a*.

**Figure 3:**
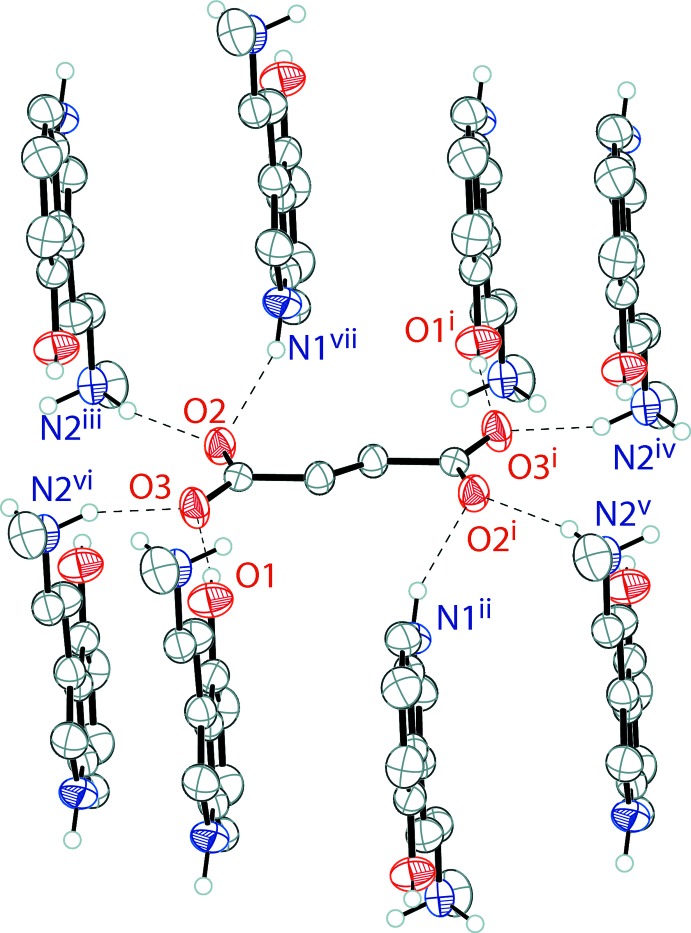
The hydrogen bonding (Table 2 ▸) of a fumarate ion in the structure of bis­(4-hy­droxy-*N*-methyl­tryptammonium)­fumarate, with hydrogen bonds shown as dashed lines. Displacement ellipsoids are drawn at the 50% probability level. Hydrogen atoms not involved in hydrogen bonding are omitted for clarity. Symmetry codes: (i) 1 − *x*, −*y*, 2 − *z*; (ii) 2 − *x*, 1 − *y*, 2 − *z*; (iii) 1 − *x*, −*y*, 1 − *z*; (iv) 2 − *x*, −*y*, 2 − *z*; (v) *x*, *y*, 1 + *z*; (vi) −1 + *x*, *y*, *z*; (vii) −1 + *x*, −1 + *y*, *z*.

**Figure 4:**
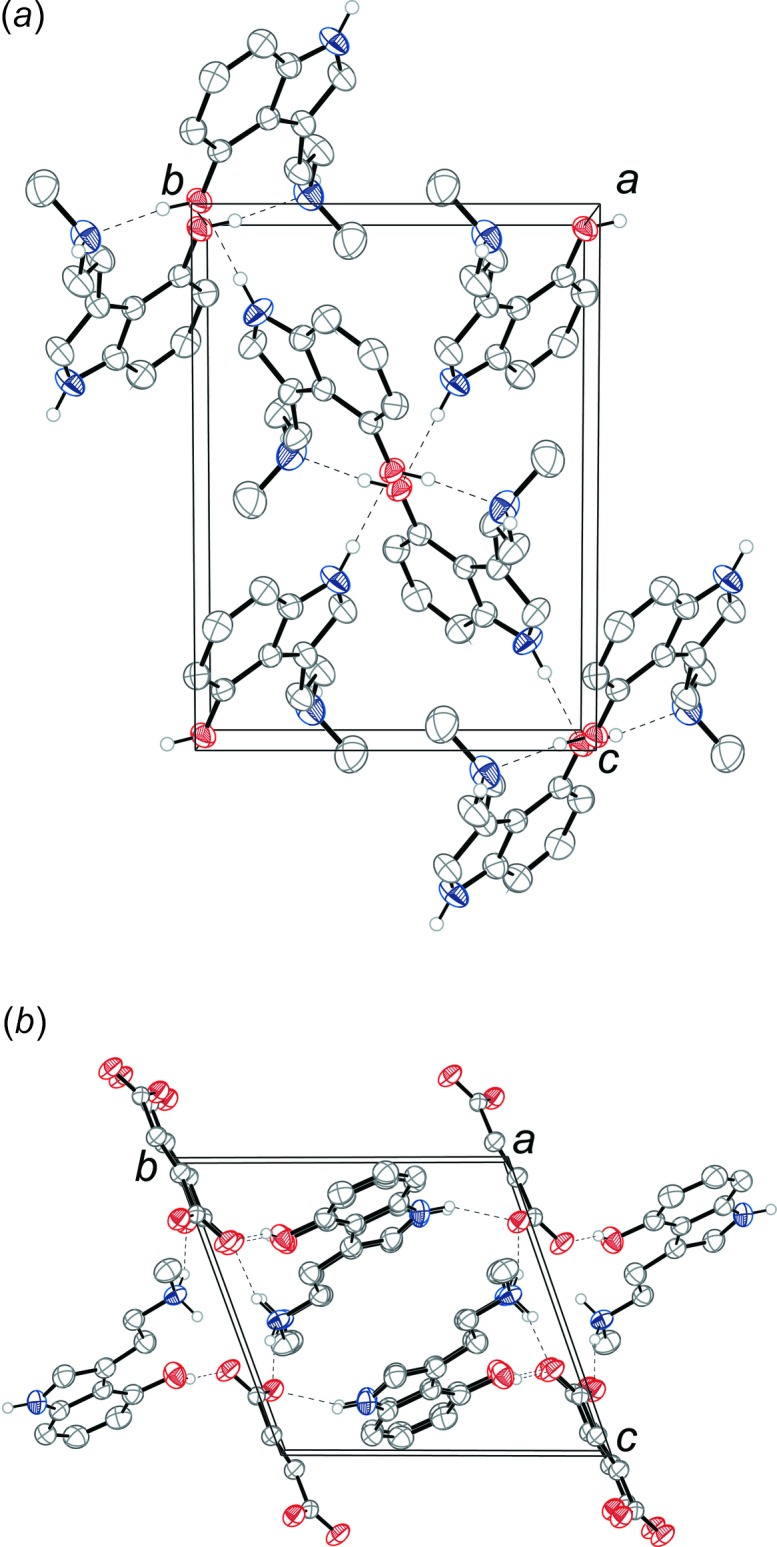
The crystal packing of (*a*) 4-HO-NMT, and of (*b*) bis­(4-HO-NMT) fumarate, both shown along the *a* axis. The hydrogen bonds (Tables 1 ▸ and 2 ▸) are shown as dashed lines. Displacement ellipsoids are drawn at the 50% probability level. Hydrogen atoms not involved in hydrogen bonding are omitted for clarity. For (*a*) only one component of the disorder is shown.

The tryptammonium cations and the fumarate dianions of the fumarate salt of norpsilocin are held together in an infinite three-dimensional framework through a series of N—H⋯O and O—H⋯O hydrogen bonds (Table 2 ▸). The indole N—H, methyl­ammonium N—H, and phenol O—H groups all hydrogen bond with the oxygen atoms of the fumarate dianion (Fig. 3 ▸). The six-membered rings of inversion-related indoles stack with parallel slipped π–π inter­actions [inter­centroid distance = 3.6465 (15) Å, inter­planar distance = 3.4781 (16) Å, and slippage = 1.095 (3) Å]. The packing of bis­(4-HO-NMT) fumarate is shown in Fig. 4 ▸ *b*.

## Database survey   

The most significant comparison to the structure of freebase norpsilocin is psilocin [CSD (Groom *et al.*, 2016 ▸) refcode PSILIN: Petcher & Weber, 1974 ▸). In the case of psilocin, the mol­ecule dimerizes through O—H⋯N hydrogen bonds, and does not form an extended network because of the lack of N—H⋯O hydrogen bonds. The other free-base tryptamines whose structures are known include natural products such as psilocybin (PSILOC: Weber & Petcher, 1974 ▸), DMT – *N*,*N*-di­methyl­tryptamine (DMTRYP: Falkenberg, 1972*b* ▸) and bufotenine (BUFTEN: Falkenberg, 1972*a* ▸), as well as synthetic tryptamines such as *N*-methyl-*N*-propyl­tryptamine (WOHYAW: Chadeayne, Golen & Manke, 2019*b* ▸).

The fumarate salt of norpsilocin crystallizes as a two-to-one tryptammonium-to-fumarate salt. This ratio has also been observed in salts of 4-acet­oxy-*N*,*N*-di­methyl­tryptammonium (XOFDOO: Chadeayne, Golen & Manke, 2019*a* ▸), 4-hy­droxy-*N*,*N*-di­propyl­tryptammonium (CCDC 1962339: Chadeayne, Pham *et al.*, 2019*b* ▸), and 4-hy­droxy-*N*-isopropyl-*N*-methyl­tryptammonium (CCDC 1987588: Chadeayne *et al.*, 2020 ▸). One-to-one tryptammonium-to-hydro­fumarate salts have been observed for 4-acet­oxy-*N*,*N*-di­methyl­tryptammonium (HOCJUH: Chadeayne *et al.*, 2019*c* ▸), 4-hy­droxy-*N*-isopropyl-*N*-methyl­tryptammonium and *N*-isopropyl-*N*-methyl­typt­ammonium (RONSUL and RONSOF: Chadeayne, Pham *et al.*, 2019*a* ▸).

## Synthesis and crystallization   

Single crystals suitable for X-ray analysis were obtained from the slow evaporation of an acetone solution of a commercial sample of 4-hy­droxy-*N*-methyl­tryptamine (Angene).

The fumarate salt was synthesized starting with 101 mg of 4-hy­droxy-*N*-methyl­tryptamine, which was dissolved in 10 mL of methanol. 62 mg of fumaric acid was added to the solution and it was stirred overnight under reflux. Solvent was removed *in vacuo* to yield a dark-blue powder. The powder was triturated with diethyl ether and then recrystallized in acetone to yield colorless crystals suitable for X-ray analysis. ^1^H NMR (400 MHz, D_2_O): δ 7.12 (*s*, 1 H, Ar*H*), 7.10–7.07 (*m*, 2 H, Ar*H*), 6.66 (*s*, 2 H, C*H*), 6.56 (*dd*, *J* = 5.5, 2.8 Hz, 1 H, Ar*H*), 3.41 (*t*, *J* = 6.8 Hz, 2 H, C*H*_2_), 3.26 (*t*, *J* = 6.8 Hz, C*H*_2_), 2.70 (*s*, 3 H, C*H*_3_); ^13^C NMR (100 MHz, D_2_O): δ 171.0 (*C*OOH), 149.7 (Ar*C*), 138.5 (Ar*C*), 134.2 (*C*H), 123.0 (Ar*C*), 122.8 (Ar*C*), 115.6 (Ar*C*), 108.4 (Ar*C*), 104.2 (Ar*C*), 103.4 (Ar*C*), 50.3 (*C*H_2_), 32.4 (*C*H_2_), 22.7 (*C*H_3_).

## Refinement   

Crystal data, data collection and structure refinement details are summarized in Table 3 ▸. Hydrogen atoms H1, H1*A*, and H2 were found from a difference-Fourier map and were refined isotropically, using DFIX restraints with N—H distances of 0.87 (1) Å and an O—H distance of 0.88 (1) Å. Isotropic displacement parameters were set to 1.2*U*_eq_ of the parent nitro­gen atom and 1.5*U*_eq_ of the parent oxygen atom. All other hydrogen atoms were placed in calculated positions (C—H = 0.93–0.97 Å). Isotropic displacement parameters were set to 1.2*U*_eq_(C) or 1.5*U*_eq_(C-meth­yl).

## Supplementary Material

Crystal structure: contains datablock(s) I, II, global. DOI: 10.1107/S2056989020004077/pk2623sup1.cif

Structure factors: contains datablock(s) I. DOI: 10.1107/S2056989020004077/pk2623Isup2.hkl

Structure factors: contains datablock(s) II. DOI: 10.1107/S2056989020004077/pk2623IIsup3.hkl

CCDC references: 1992279, 1992278

Additional supporting information:  crystallographic information; 3D view; checkCIF report

## Figures and Tables

**Table 1:** Hydrogen-bond geometry (Å, °) for (I)

*D*—H⋯*A*	*D*—H	H⋯*A*	*D*⋯*A*	*D*—H⋯*A*
O1—H1⋯N2^i^	0.86 (1)	1.80 (1)	2.6501 (16)	169 (2)
N1—H1*A*⋯O1^ii^	0.88 (1)	2.04 (1)	2.9092 (15)	175 (2)

Symmetry codes: (i) 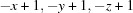; (ii) 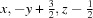.

**Table 2:** Hydrogen-bond geometry (Å, °) for (II)

*D*—H⋯*A*	*D*—H	H⋯*A*	*D*⋯*A*	*D*—H⋯*A*
O1—H1⋯O3	0.87 (1)	1.89 (1)	2.7399 (16)	163 (2)
N1—H1*A*⋯O2^i^	0.86 (1)	2.07 (1)	2.8854 (18)	157 (2)
N2—H2*A*⋯O3^ii^	0.89 (1)	1.90 (1)	2.7349 (18)	155 (2)
N2—H2*B*⋯O2^iii^	0.89 (1)	1.91 (1)	2.7715 (19)	164 (2)

Symmetry codes: (i) 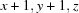; (ii) 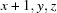; (iii) 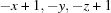.

**Table 3:** Experimental details

	(I)	(II)
Crystal data		
Chemical formula	C_11_H_14_N_2_O	C_11_H_15_N_2_O^+^·0.5C_4_H_2_O_4_^2−^
*M*_r_	190.24	248.28
Crystal system, space group	Monoclinic, *P*2_1_/*c*	Triclinic, *P* 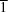
Temperature (K)	297	297
*a*, *b*, *c* (Å)	9.4060 (16), 8.8436 (15), 12.144 (2)	7.7363 (10), 9.7146 (12), 9.7854 (13)
α, β, γ (°)	90, 100.601 (7), 90	105.524 (4), 110.554 (4), 97.167 (4)
*V* (Å^3^)	993.0 (3)	643.69 (14)
*Z*	4	2
Radiation type	Mo *K*α	Mo *K*α
μ (mm^−1^)	0.08	0.09
Crystal size (mm)	0.35 × 0.2 × 0.1	0.24 × 0.19 × 0.03

Data collection		
Diffractometer	Bruker D8 Venture CMOS	Bruker D8 Venture CMOS
Absorption correction	Multi-scan (*SADABS*; Bruker, 2018 ▸)	Multi-scan (*SADABS*; Bruker, 2018 ▸)
*T*_min_, *T*_max_	0.716, 0.745	0.685, 0.745
No. of measured, independent and observed [*I* > 2σ(*I*)] reflections	35681, 1955, 1687	14395, 2365, 1774
*R*_int_	0.031	0.046
(sin θ/λ)_max_ (Å^−1^)	0.620	0.605

Refinement		
*R*[*F* ^2^ > 2σ(*F* ^2^)], *wR*(*F* ^2^), *S*	0.038, 0.105, 1.09	0.039, 0.098, 1.11
No. of reflections	1955	2365
No. of parameters	171	181
No. of restraints	105	4
H-atom treatment	H atoms treated by a mixture of independent and constrained refinement	H atoms treated by a mixture of independent and constrained refinement
Δρ_max_, Δρ_min_ (e Å^−3^)	0.20, −0.14	0.15, −0.15

Computer programs: *APEX3* and *SAINT* (Bruker, 2018 ▸), SHELXT2014 (Sheldrick, 2015*a* ▸), *SHELXL2018* (Sheldrick, 2015*b* ▸), *OLEX2* (Dolomanov *et al.*, 2009 ▸) and *publCIF* (Westrip, 2010 ▸).

## supplementary crystallographic information

### 3-[2-(Methylamino)ethyl]-1H-indol-4-ol (I) Crystal data

**Table:**

C_11_H_14_N_2_O	*F*(000) = 408
*M**_r_* = 190.24	*D*_x_ = 1.273 Mg m^−^^3^
Monoclinic, *P*2_1_/*c*	Mo *K*α radiation, λ = 0.71073 Å
*a* = 9.4060 (16) Å	Cell parameters from 9944 reflections
*b* = 8.8436 (15) Å	θ = 2.9–26.0°
*c* = 12.144 (2) Å	µ = 0.08 mm^−^^1^
β = 100.601 (7)°	*T* = 297 K
*V* = 993.0 (3) Å^3^	BLOCK, colourless
*Z* = 4	0.35 × 0.2 × 0.1 mm

### 3-[2-(Methylamino)ethyl]-1H-indol-4-ol (I) Data collection

**Table:**

Bruker D8 Venture CMOS diffractometer	1687 reflections with *I* > 2σ(*I*)
φ and ω scans	*R*_int_ = 0.031
Absorption correction: multi-scan (SADABS; Bruker, 2018)	θ_max_ = 26.1°, θ_min_ = 3.2°
*T*_min_ = 0.716, *T*_max_ = 0.745	*h* = −11→11
35681 measured reflections	*k* = −10→10
1955 independent reflections	*l* = −14→15

### 3-[2-(Methylamino)ethyl]-1H-indol-4-ol (I) Refinement

**Table:**

Refinement on *F*^2^	105 restraints
Least-squares matrix: full	Hydrogen site location: mixed
*R*[*F*^2^ > 2σ(*F*^2^)] = 0.038	H atoms treated by a mixture of independent and constrained refinement
*wR*(*F*^2^) = 0.105	*w* = 1/[σ^2^(*F*_o_^2^) + (0.050*P*)^2^ + 0.2586*P*] where *P* = (*F*_o_^2^ + 2*F*_c_^2^)/3
*S* = 1.09	(Δ/σ)_max_ < 0.001
1955 reflections	Δρ_max_ = 0.20 e Å^−^^3^
171 parameters	Δρ_min_ = −0.14 e Å^−^^3^

### 3-[2-(Methylamino)ethyl]-1H-indol-4-ol (I) Special details

**Table:**

Geometry. All esds (except the esd in the dihedral angle between two l.s. planes) are estimated using the full covariance matrix. The cell esds are taken into account individually in the estimation of esds in distances, angles and torsion angles; correlations between esds in cell parameters are only used when they are defined by crystal symmetry. An approximate (isotropic) treatment of cell esds is used for estimating esds involving l.s. planes.

### 3-[2-(Methylamino)ethyl]-1H-indol-4-ol (I) Fractional atomic coordinates and isotropic or equivalent isotropic displacement parameters (Å^2^)

**Table:**

	*x*	*y*	*z*	*U*_iso_*/*U*_eq_	Occ. (<1)
O1	0.72180 (10)	0.50889 (10)	0.48264 (7)	0.0392 (3)	
H1	0.7503 (17)	0.4189 (12)	0.5019 (14)	0.059*	
N1	0.67378 (13)	0.84618 (14)	0.18771 (10)	0.0460 (3)	
H1A	0.6832 (18)	0.8918 (18)	0.1255 (10)	0.055*	
C1	0.70586 (13)	0.68167 (13)	0.33046 (10)	0.0311 (3)	
C2	0.77773 (13)	0.56216 (13)	0.39398 (9)	0.0313 (3)	
C3	0.90077 (14)	0.50210 (15)	0.36388 (11)	0.0387 (3)	
H3	0.949534	0.424117	0.406353	0.046*	
C4	0.95365 (15)	0.55642 (17)	0.27050 (12)	0.0451 (3)	
H4	1.036369	0.513198	0.252150	0.054*	
C5	0.88612 (15)	0.67154 (16)	0.20592 (12)	0.0441 (3)	
H5	0.920870	0.707054	0.143824	0.053*	
C6	0.76258 (14)	0.73365 (14)	0.23720 (10)	0.0364 (3)	
C7	0.56146 (15)	0.86403 (16)	0.24481 (12)	0.0440 (3)	
H7	0.486086	0.932698	0.225640	0.053*	
C8	0.57603 (14)	0.76694 (14)	0.33354 (11)	0.0356 (3)	
C9	0.4767 (3)	0.7551 (3)	0.4179 (3)	0.0401 (6)	0.895 (3)
H9A	0.512632	0.820479	0.481067	0.048*	0.895 (3)
H9B	0.478389	0.652079	0.445479	0.048*	0.895 (3)
C10	0.32164 (18)	0.7988 (2)	0.36920 (16)	0.0426 (4)	0.895 (3)
H10A	0.318567	0.904813	0.348540	0.051*	0.895 (3)
H10B	0.288874	0.740378	0.301680	0.051*	0.895 (3)
N2	0.22260 (16)	0.77279 (16)	0.44818 (14)	0.0414 (4)	0.895 (3)
H2	0.1338 (10)	0.778 (2)	0.4114 (8)	0.050*	0.895 (3)
C11	0.2400 (3)	0.8822 (2)	0.54022 (17)	0.0644 (6)	0.895 (3)
H11A	0.233857	0.982920	0.510261	0.097*	0.895 (3)
H11B	0.332532	0.867982	0.587682	0.097*	0.895 (3)
H11C	0.164866	0.867360	0.583032	0.097*	0.895 (3)
C9'	0.477 (3)	0.724 (3)	0.410 (3)	0.0401 (6)	0.105 (3)
H9'A	0.533638	0.706474	0.484119	0.048*	0.105 (3)
H9'B	0.430684	0.628565	0.384330	0.048*	0.105 (3)
C10'	0.3608 (18)	0.8382 (19)	0.4191 (15)	0.050 (2)	0.105 (3)
H10C	0.405248	0.936642	0.435346	0.060*	0.105 (3)
H10D	0.294887	0.844927	0.347824	0.060*	0.105 (3)
N2'	0.2784 (14)	0.7990 (17)	0.5074 (13)	0.053 (3)	0.105 (3)
H2'	0.256 (4)	0.703 (2)	0.500 (4)	0.063*	0.105 (3)
C11'	0.143 (2)	0.884 (3)	0.507 (2)	0.083 (6)	0.105 (3)
H11D	0.098734	0.849865	0.567605	0.125*	0.105 (3)
H11E	0.165025	0.990058	0.516272	0.125*	0.105 (3)
H11F	0.078492	0.868185	0.437255	0.125*	0.105 (3)

### 3-[2-(Methylamino)ethyl]-1H-indol-4-ol (I) Atomic displacement parameters (Å^2^)

**Table:**

	*U*^11^	*U*^22^	*U*^33^	*U*^12^	*U*^13^	*U*^23^
O1	0.0534 (6)	0.0345 (5)	0.0337 (5)	0.0108 (4)	0.0185 (4)	0.0063 (4)
N1	0.0523 (7)	0.0466 (7)	0.0415 (6)	0.0002 (5)	0.0151 (5)	0.0174 (5)
C1	0.0332 (6)	0.0300 (6)	0.0311 (6)	−0.0049 (5)	0.0081 (5)	−0.0009 (4)
C2	0.0354 (6)	0.0311 (6)	0.0279 (6)	−0.0023 (5)	0.0074 (4)	−0.0021 (4)
C3	0.0362 (7)	0.0393 (7)	0.0407 (7)	0.0042 (5)	0.0074 (5)	0.0000 (5)
C4	0.0368 (7)	0.0494 (8)	0.0536 (8)	−0.0024 (6)	0.0199 (6)	−0.0049 (6)
C5	0.0448 (7)	0.0475 (8)	0.0451 (7)	−0.0096 (6)	0.0218 (6)	0.0017 (6)
C6	0.0385 (7)	0.0359 (6)	0.0357 (6)	−0.0072 (5)	0.0097 (5)	0.0025 (5)
C7	0.0448 (7)	0.0419 (7)	0.0463 (7)	0.0058 (6)	0.0109 (6)	0.0132 (6)
C8	0.0372 (6)	0.0334 (6)	0.0374 (6)	0.0012 (5)	0.0097 (5)	0.0051 (5)
C9	0.0448 (7)	0.0372 (15)	0.0414 (10)	0.0089 (9)	0.0160 (6)	0.0076 (10)
C10	0.0412 (9)	0.0473 (10)	0.0411 (9)	0.0100 (7)	0.0124 (7)	0.0124 (7)
N2	0.0383 (7)	0.0431 (8)	0.0450 (8)	0.0085 (6)	0.0132 (6)	0.0071 (6)
C11	0.0799 (15)	0.0521 (11)	0.0699 (13)	0.0053 (11)	0.0368 (11)	−0.0077 (10)
C9'	0.0448 (7)	0.0372 (15)	0.0414 (10)	0.0089 (9)	0.0160 (6)	0.0076 (10)
C10'	0.053 (5)	0.046 (4)	0.053 (5)	0.007 (4)	0.015 (4)	0.005 (4)
N2'	0.059 (5)	0.046 (5)	0.059 (6)	0.007 (5)	0.026 (5)	0.001 (5)
C11'	0.066 (10)	0.078 (11)	0.112 (13)	0.024 (9)	0.038 (9)	0.027 (10)

### 3-[2-(Methylamino)ethyl]-1H-indol-4-ol (I) Geometric parameters (Å, º)

**Table:**

O1—H1	0.858 (9)	C9—C10	1.520 (3)
O1—C2	1.3662 (14)	C10—H10A	0.9700
N1—H1A	0.875 (9)	C10—H10B	0.9700
N1—C6	1.3655 (18)	C10—N2	1.4729 (19)
N1—C7	1.3753 (18)	N2—H2	0.873 (9)
C1—C2	1.4061 (17)	N2—C11	1.464 (2)
C1—C6	1.4147 (17)	C11—H11A	0.9600
C1—C8	1.4416 (17)	C11—H11B	0.9600
C2—C3	1.3823 (18)	C11—H11C	0.9600
C3—H3	0.9300	C9'—H9'A	0.9700
C3—C4	1.4042 (19)	C9'—H9'B	0.9700
C4—H4	0.9300	C9'—C10'	1.509 (10)
C4—C5	1.368 (2)	C10'—H10C	0.9700
C5—H5	0.9300	C10'—H10D	0.9700
C5—C6	1.3995 (19)	C10'—N2'	1.476 (9)
C7—H7	0.9300	N2'—H2'	0.876 (14)
C7—C8	1.3649 (18)	N2'—C11'	1.476 (10)
C8—C9	1.512 (3)	C11'—H11D	0.9600
C8—C9'	1.48 (3)	C11'—H11E	0.9600
C9—H9A	0.9700	C11'—H11F	0.9600
C9—H9B	0.9700		
			
C2—O1—H1	112.8 (11)	C9—C10—H10A	109.1
C6—N1—H1A	124.4 (12)	C9—C10—H10B	109.1
C6—N1—C7	109.04 (11)	H10A—C10—H10B	107.8
C7—N1—H1A	126.3 (11)	N2—C10—C9	112.57 (17)
C2—C1—C6	118.00 (11)	N2—C10—H10A	109.1
C2—C1—C8	134.67 (11)	N2—C10—H10B	109.1
C6—C1—C8	107.27 (11)	C10—N2—H2	108.6 (7)
O1—C2—C1	118.40 (10)	C11—N2—C10	113.51 (15)
O1—C2—C3	122.57 (11)	C11—N2—H2	108.6 (7)
C3—C2—C1	119.03 (11)	N2—C11—H11A	109.5
C2—C3—H3	119.3	N2—C11—H11B	109.5
C2—C3—C4	121.35 (13)	N2—C11—H11C	109.5
C4—C3—H3	119.3	H11A—C11—H11B	109.5
C3—C4—H4	119.3	H11A—C11—H11C	109.5
C5—C4—C3	121.43 (13)	H11B—C11—H11C	109.5
C5—C4—H4	119.3	C8—C9'—H9'A	108.6
C4—C5—H5	121.4	C8—C9'—H9'B	108.6
C4—C5—C6	117.23 (12)	C8—C9'—C10'	115 (2)
C6—C5—H5	121.4	H9'A—C9'—H9'B	107.6
N1—C6—C1	107.40 (11)	C10'—C9'—H9'A	108.6
N1—C6—C5	129.63 (12)	C10'—C9'—H9'B	108.6
C5—C6—C1	122.96 (12)	C9'—C10'—H10C	109.1
N1—C7—H7	124.7	C9'—C10'—H10D	109.1
C8—C7—N1	110.55 (12)	H10C—C10'—H10D	107.9
C8—C7—H7	124.7	N2'—C10'—C9'	112.3 (16)
C1—C8—C9	127.83 (14)	N2'—C10'—H10C	109.1
C1—C8—C9'	120.9 (8)	N2'—C10'—H10D	109.1
C7—C8—C1	105.73 (11)	C10'—N2'—H2'	107.7 (14)
C7—C8—C9	126.43 (15)	C10'—N2'—C11'	116.4 (13)
C7—C8—C9'	132.4 (9)	C11'—N2'—H2'	107.6 (14)
C8—C9—H9A	109.0	N2'—C11'—H11D	109.5
C8—C9—H9B	109.0	N2'—C11'—H11E	109.5
C8—C9—C10	112.8 (2)	N2'—C11'—H11F	109.5
H9A—C9—H9B	107.8	H11D—C11'—H11E	109.5
C10—C9—H9A	109.0	H11D—C11'—H11F	109.5
C10—C9—H9B	109.0	H11E—C11'—H11F	109.5
			
O1—C2—C3—C4	178.26 (11)	C6—C1—C2—O1	−178.50 (10)
N1—C7—C8—C1	−0.66 (16)	C6—C1—C2—C3	0.80 (17)
N1—C7—C8—C9	178.33 (19)	C6—C1—C8—C7	−0.28 (14)
N1—C7—C8—C9'	−169 (2)	C6—C1—C8—C9	−179.24 (18)
C1—C2—C3—C4	−1.01 (19)	C6—C1—C8—C9'	169.9 (18)
C1—C8—C9—C10	−151.89 (16)	C7—N1—C6—C1	−1.52 (15)
C1—C8—C9'—C10'	169.5 (18)	C7—N1—C6—C5	176.94 (14)
C2—C1—C6—N1	178.58 (11)	C7—C8—C9—C10	29.3 (3)
C2—C1—C6—C5	−0.01 (19)	C7—C8—C9'—C10'	−23 (4)
C2—C1—C8—C7	−177.14 (14)	C8—C1—C2—O1	−1.9 (2)
C2—C1—C8—C9	3.9 (3)	C8—C1—C2—C3	177.41 (13)
C2—C1—C8—C9'	−7.0 (18)	C8—C1—C6—N1	1.11 (14)
C2—C3—C4—C5	0.4 (2)	C8—C1—C6—C5	−177.48 (12)
C3—C4—C5—C6	0.4 (2)	C8—C9—C10—N2	174.34 (15)
C4—C5—C6—N1	−178.84 (14)	C8—C9'—C10'—N2'	−172 (2)
C4—C5—C6—C1	−0.6 (2)	C9—C10—N2—C11	73.1 (2)
C6—N1—C7—C8	1.39 (17)	C9'—C10'—N2'—C11'	−167 (3)

### 3-[2-(Methylamino)ethyl]-1H-indol-4-ol (I) Hydrogen-bond geometry (Å, º)

**Table:**

*D*—H···*A*	*D*—H	H···*A*	*D*···*A*	*D*—H···*A*
O1—H1···N2^i^	0.86 (1)	1.80 (1)	2.6501 (16)	169 (2)
N1—H1*A*···O1^ii^	0.88 (1)	2.04 (1)	2.9092 (15)	175 (2)

Symmetry codes: (i) −*x*+1, −*y*+1, −*z*+1; (ii) *x*, −*y*+3/2, *z*−1/2.

### Bis{[2-(4-hydroxy-1H-indol-3-yl)ethyl]methylazanium} but-2-enedioate (II) Crystal data

**Table:**

C_11_H_15_N_2_O^+^·0.5C_4_H_2_O_4_^2^^−^	*Z* = 2
*M**_r_* = 248.28	*F*(000) = 264
Triclinic, *P*1	*D*_x_ = 1.281 Mg m^−^^3^
*a* = 7.7363 (10) Å	Mo *K*α radiation, λ = 0.71073 Å
*b* = 9.7146 (12) Å	Cell parameters from 3848 reflections
*c* = 9.7854 (13) Å	θ = 2.7–25.5°
α = 105.524 (4)°	µ = 0.09 mm^−^^1^
β = 110.554 (4)°	*T* = 297 K
γ = 97.167 (4)°	BLOCK, colourless
*V* = 643.69 (14) Å^3^	0.24 × 0.19 × 0.03 mm

### Bis{[2-(4-hydroxy-1H-indol-3-yl)ethyl]methylazanium} but-2-enedioate (II) Data collection

**Table:**

Bruker D8 Venture CMOS diffractometer	1774 reflections with *I* > 2σ(*I*)
φ and ω scans	*R*_int_ = 0.046
Absorption correction: multi-scan (SADABS; Bruker, 2018)	θ_max_ = 25.5°, θ_min_ = 2.7°
*T*_min_ = 0.685, *T*_max_ = 0.745	*h* = −9→9
14395 measured reflections	*k* = −11→11
2365 independent reflections	*l* = −11→11

### Bis{[2-(4-hydroxy-1H-indol-3-yl)ethyl]methylazanium} but-2-enedioate (II) Refinement

**Table:**

Refinement on *F*^2^	Hydrogen site location: mixed
Least-squares matrix: full	H atoms treated by a mixture of independent and constrained refinement
*R*[*F*^2^ > 2σ(*F*^2^)] = 0.039	*w* = 1/[σ^2^(*F*_o_^2^) + (0.0428*P*)^2^ + 0.1031*P*] where *P* = (*F*_o_^2^ + 2*F*_c_^2^)/3
*wR*(*F*^2^) = 0.098	(Δ/σ)_max_ < 0.001
*S* = 1.11	Δρ_max_ = 0.15 e Å^−^^3^
2365 reflections	Δρ_min_ = −0.15 e Å^−^^3^
181 parameters	Extinction correction: SHELXL2018 (Sheldrick, 2015b), Fc^*^=kFc[1+0.001xFc^2^λ^3^/sin(2θ)]^-1/4^
4 restraints	Extinction coefficient: 0.035 (8)

### Bis{[2-(4-hydroxy-1H-indol-3-yl)ethyl]methylazanium} but-2-enedioate (II) Special details

**Table:**

Geometry. All esds (except the esd in the dihedral angle between two l.s. planes) are estimated using the full covariance matrix. The cell esds are taken into account individually in the estimation of esds in distances, angles and torsion angles; correlations between esds in cell parameters are only used when they are defined by crystal symmetry. An approximate (isotropic) treatment of cell esds is used for estimating esds involving l.s. planes.

### Bis{[2-(4-hydroxy-1H-indol-3-yl)ethyl]methylazanium} but-2-enedioate (II) Fractional atomic coordinates and isotropic or equivalent isotropic displacement parameters (Å^2^)

**Table:**

	*x*	*y*	*z*	*U*_iso_*/*U*_eq_	
O1	0.71021 (18)	0.24171 (13)	0.73074 (16)	0.0516 (4)	
N1	1.1025 (2)	0.68290 (16)	0.82025 (18)	0.0458 (4)	
N2	1.1755 (2)	0.13836 (16)	0.45820 (17)	0.0390 (4)	
C1	0.9034 (2)	0.47406 (17)	0.77976 (18)	0.0351 (4)	
C2	0.7537 (2)	0.39203 (18)	0.79685 (19)	0.0391 (4)	
C3	0.6597 (3)	0.4650 (2)	0.8775 (2)	0.0492 (5)	
H3	0.559823	0.411333	0.888131	0.059*	
C4	0.7117 (3)	0.6188 (2)	0.9441 (2)	0.0532 (5)	
H4	0.644580	0.664964	0.997328	0.064*	
C5	0.8583 (3)	0.7030 (2)	0.9328 (2)	0.0480 (5)	
H5	0.893452	0.804937	0.978251	0.058*	
C6	0.9524 (2)	0.62851 (18)	0.85004 (19)	0.0391 (4)	
C7	1.1455 (3)	0.56779 (19)	0.7313 (2)	0.0444 (4)	
H7	1.240746	0.577663	0.694858	0.053*	
C8	1.0291 (2)	0.43736 (18)	0.70422 (19)	0.0379 (4)	
C9	1.0327 (3)	0.28658 (18)	0.6159 (2)	0.0434 (4)	
H9A	0.906459	0.236367	0.536418	0.052*	
H9B	1.066169	0.230581	0.685761	0.052*	
C10	1.1729 (2)	0.29082 (18)	0.5407 (2)	0.0398 (4)	
H10A	1.137504	0.343594	0.467796	0.048*	
H10B	1.299028	0.342809	0.619295	0.048*	
C11	1.3055 (3)	0.1317 (2)	0.3774 (2)	0.0561 (5)	
H11A	1.293563	0.030805	0.321818	0.084*	
H11B	1.434183	0.174879	0.451983	0.084*	
H11C	1.273049	0.184976	0.306086	0.084*	
C12	0.3434 (2)	0.02114 (16)	0.80503 (17)	0.0311 (4)	
C13	0.5073 (2)	0.01566 (17)	0.94080 (18)	0.0344 (4)	
H13	0.628166	0.035974	0.941097	0.041*	
O2	0.17836 (15)	−0.03196 (13)	0.78734 (13)	0.0423 (3)	
O3	0.38449 (16)	0.07932 (13)	0.71675 (14)	0.0461 (3)	
H1	0.613 (2)	0.203 (2)	0.745 (3)	0.074 (7)*	
H1A	1.141 (3)	0.7760 (12)	0.839 (2)	0.069 (7)*	
H2A	1.216 (3)	0.0955 (19)	0.5298 (18)	0.054 (6)*	
H2B	1.0586 (16)	0.0926 (19)	0.3889 (18)	0.054 (6)*	

### Bis{[2-(4-hydroxy-1H-indol-3-yl)ethyl]methylazanium} but-2-enedioate (II) Atomic displacement parameters (Å^2^)

**Table:**

	*U*^11^	*U*^22^	*U*^33^	*U*^12^	*U*^13^	*U*^23^
O1	0.0481 (8)	0.0429 (7)	0.0624 (9)	−0.0009 (6)	0.0278 (7)	0.0135 (6)
N1	0.0510 (9)	0.0347 (8)	0.0500 (9)	0.0033 (7)	0.0194 (8)	0.0161 (7)
N2	0.0369 (8)	0.0476 (9)	0.0337 (8)	0.0135 (7)	0.0111 (7)	0.0178 (7)
C1	0.0342 (9)	0.0385 (9)	0.0306 (9)	0.0070 (7)	0.0094 (7)	0.0145 (7)
C2	0.0376 (9)	0.0407 (9)	0.0353 (9)	0.0049 (7)	0.0115 (8)	0.0133 (7)
C3	0.0429 (10)	0.0626 (12)	0.0477 (11)	0.0107 (9)	0.0233 (9)	0.0212 (9)
C4	0.0609 (12)	0.0589 (12)	0.0486 (12)	0.0230 (10)	0.0304 (10)	0.0167 (9)
C5	0.0625 (12)	0.0408 (10)	0.0409 (10)	0.0169 (9)	0.0199 (9)	0.0130 (8)
C6	0.0419 (9)	0.0393 (9)	0.0348 (9)	0.0079 (7)	0.0114 (8)	0.0166 (7)
C7	0.0433 (10)	0.0475 (10)	0.0468 (11)	0.0067 (8)	0.0217 (9)	0.0199 (8)
C8	0.0369 (9)	0.0417 (9)	0.0363 (9)	0.0085 (7)	0.0139 (8)	0.0162 (7)
C9	0.0430 (10)	0.0425 (10)	0.0457 (10)	0.0084 (8)	0.0201 (9)	0.0144 (8)
C10	0.0387 (9)	0.0421 (9)	0.0389 (10)	0.0102 (7)	0.0132 (8)	0.0170 (7)
C11	0.0576 (12)	0.0740 (14)	0.0527 (12)	0.0281 (10)	0.0307 (10)	0.0291 (10)
C12	0.0330 (9)	0.0272 (8)	0.0294 (8)	0.0041 (6)	0.0086 (7)	0.0106 (6)
C13	0.0287 (8)	0.0385 (9)	0.0343 (9)	0.0043 (7)	0.0097 (7)	0.0154 (7)
O2	0.0297 (6)	0.0494 (7)	0.0429 (7)	0.0014 (5)	0.0060 (5)	0.0233 (6)
O3	0.0399 (7)	0.0606 (8)	0.0412 (7)	0.0057 (6)	0.0114 (6)	0.0322 (6)

### Bis{[2-(4-hydroxy-1H-indol-3-yl)ethyl]methylazanium} but-2-enedioate (II) Geometric parameters (Å, º)

**Table:**

O1—C2	1.372 (2)	C5—C6	1.393 (3)
O1—H1	0.870 (10)	C7—H7	0.9300
N1—C6	1.372 (2)	C7—C8	1.362 (2)
N1—C7	1.376 (2)	C8—C9	1.498 (2)
N1—H1A	0.863 (10)	C9—H9A	0.9700
N2—C10	1.492 (2)	C9—H9B	0.9700
N2—C11	1.479 (2)	C9—C10	1.511 (2)
N2—H2A	0.892 (9)	C10—H10A	0.9700
N2—H2B	0.885 (9)	C10—H10B	0.9700
C1—C2	1.408 (2)	C11—H11A	0.9600
C1—C6	1.411 (2)	C11—H11B	0.9600
C1—C8	1.438 (2)	C11—H11C	0.9600
C2—C3	1.373 (3)	C12—C13	1.499 (2)
C3—H3	0.9300	C12—O2	1.2488 (18)
C3—C4	1.402 (3)	C12—O3	1.2553 (18)
C4—H4	0.9300	C13—C13^i^	1.311 (3)
C4—C5	1.368 (3)	C13—H13	0.9300
C5—H5	0.9300		
			
C2—O1—H1	109.6 (15)	C8—C7—N1	110.51 (15)
C6—N1—C7	108.98 (14)	C8—C7—H7	124.7
C6—N1—H1A	121.5 (15)	C1—C8—C9	127.04 (14)
C7—N1—H1A	128.2 (15)	C7—C8—C1	105.81 (15)
C10—N2—H2A	106.9 (13)	C7—C8—C9	127.14 (16)
C10—N2—H2B	107.9 (13)	C8—C9—H9A	109.1
C11—N2—C10	114.20 (14)	C8—C9—H9B	109.1
C11—N2—H2A	107.5 (13)	C8—C9—C10	112.38 (13)
C11—N2—H2B	108.4 (13)	H9A—C9—H9B	107.9
H2A—N2—H2B	112.1 (18)	C10—C9—H9A	109.1
C2—C1—C6	117.87 (15)	C10—C9—H9B	109.1
C2—C1—C8	134.53 (15)	N2—C10—C9	110.39 (13)
C6—C1—C8	107.60 (14)	N2—C10—H10A	109.6
O1—C2—C1	117.25 (15)	N2—C10—H10B	109.6
O1—C2—C3	123.67 (15)	C9—C10—H10A	109.6
C3—C2—C1	119.08 (16)	C9—C10—H10B	109.6
C2—C3—H3	119.4	H10A—C10—H10B	108.1
C2—C3—C4	121.20 (17)	N2—C11—H11A	109.5
C4—C3—H3	119.4	N2—C11—H11B	109.5
C3—C4—H4	119.1	N2—C11—H11C	109.5
C5—C4—C3	121.82 (18)	H11A—C11—H11B	109.5
C5—C4—H4	119.1	H11A—C11—H11C	109.5
C4—C5—H5	121.6	H11B—C11—H11C	109.5
C4—C5—C6	116.76 (17)	O2—C12—C13	118.55 (13)
C6—C5—H5	121.6	O2—C12—O3	124.96 (14)
N1—C6—C1	107.08 (15)	O3—C12—C13	116.49 (14)
N1—C6—C5	129.66 (16)	C12—C13—H13	117.6
C5—C6—C1	123.26 (16)	C13^i^—C13—C12	124.77 (19)
N1—C7—H7	124.7	C13^i^—C13—H13	117.6
			
O1—C2—C3—C4	179.30 (17)	C6—C1—C2—C3	1.4 (2)
N1—C7—C8—C1	0.79 (19)	C6—C1—C8—C7	−0.02 (18)
N1—C7—C8—C9	−178.45 (16)	C6—C1—C8—C9	179.22 (16)
C1—C2—C3—C4	−0.7 (3)	C7—N1—C6—C1	1.23 (18)
C1—C8—C9—C10	175.12 (15)	C7—N1—C6—C5	−179.00 (18)
C2—C1—C6—N1	178.79 (14)	C7—C8—C9—C10	−5.8 (3)
C2—C1—C6—C5	−1.0 (2)	C8—C1—C2—O1	0.7 (3)
C2—C1—C8—C7	−179.45 (18)	C8—C1—C2—C3	−179.26 (18)
C2—C1—C8—C9	−0.2 (3)	C8—C1—C6—N1	−0.74 (18)
C2—C3—C4—C5	−0.5 (3)	C8—C1—C6—C5	179.47 (16)
C3—C4—C5—C6	0.8 (3)	C8—C9—C10—N2	178.34 (14)
C4—C5—C6—N1	−179.84 (17)	C11—N2—C10—C9	178.49 (15)
C4—C5—C6—C1	−0.1 (3)	O2—C12—C13—C13^i^	−13.3 (3)
C6—N1—C7—C8	−1.3 (2)	O3—C12—C13—C13^i^	167.0 (2)
C6—C1—C2—O1	−178.63 (14)		

Symmetry code: (i) −*x*+1, −*y*, −*z*+2.

### Bis{[2-(4-hydroxy-1H-indol-3-yl)ethyl]methylazanium} but-2-enedioate (II) Hydrogen-bond geometry (Å, º)

**Table:**

*D*—H···*A*	*D*—H	H···*A*	*D*···*A*	*D*—H···*A*
O1—H1···O3	0.87 (1)	1.89 (1)	2.7399 (16)	163 (2)
N1—H1*A*···O2^ii^	0.86 (1)	2.07 (1)	2.8854 (18)	157 (2)
N2—H2*A*···O3^iii^	0.89 (1)	1.90 (1)	2.7349 (18)	155 (2)
N2—H2*B*···O2^iv^	0.89 (1)	1.91 (1)	2.7715 (19)	164 (2)

Symmetry codes: (ii) *x*+1, *y*+1, *z*; (iii) *x*+1, *y*, *z*; (iv) −*x*+1, −*y*, −*z*+1.
